# Preadmission Statin Therapy Is Associated with a Lower Incidence of Acute Kidney Injury in Critically Ill Patients: A Retrospective Observational Study

**DOI:** 10.3390/jcm8010025

**Published:** 2018-12-25

**Authors:** Tak Kyu Oh, In-Ae Song, Young-Jae Cho, Cheong Lim, Young-Tae Jeon, Hee-Joon Bae, You Hwan Jo

**Affiliations:** 1 Department of Anesthesiology and Pain Medicine, Seoul National University Bundang Hospital, Gumi-ro 173 Beon-gil, Bundang-gu, Seongnam 13620, Korea; songoficu@outlook.kr (I.-A.S.); ytjeon@snubh.org (Y.-T.J.); 2 Division of Pulmonary and Critical Care Medicine, Department of Internal Medicine, Seoul National University Bundang Hospital, Gumi-ro 173 Beon-gil, Bundang-gu, Seongnam 13620, Korea; lungdrcho@snubh.org; 3 Department of Thoracic and Cardiovascular Surgery, Seoul National University Bundang Hospital, Gumi-ro 173 Beon-gil, Bundang-gu, Seongnam 13620, Korea; mluemoon@snubh.org; 4 Department of Neurology, Stroke Center, Seoul National University Bundang Hospital, Gumi-ro 173 Beon-gil, Bundang-gu, Seongnam 13620, Korea; braindoc@snubh.org; 5 Department of Emergency Medicine, Seoul National University Bundang Hospital, Gumi-ro 173 Beon-gil, Bundang-gu, Seongnam 13620, Korea; drakejo@snubh.org

**Keywords:** acute kidney injury, statins, chronic kidney disease

## Abstract

This study aimed to investigate the association between preadmission statin use and acute kidney injury (AKI) incidence among critically ill patients who needed admission to the intensive care unit (ICU) for medical care. Medical records of patients admitted to the ICU were reviewed. Patients who continuously took statin for >1 month prior to ICU admission were defined as statin users. We investigated whether preadmission statin use was associated with AKI incidence within 72 h after ICU admission and whether the association differs according to preadmission estimated glomerular filtration rate (eGFR; in mL min^−1^ 1.73 m^−2^). Among 21,236 patients examined, 5756 (27.1%) were preadmission statin users and 15,480 (72.9%) were non-statin users. Total AKI incidence within 72 h after ICU admission was 31% lower in preadmission statin users than in non-statin users [odds ratio (OR), 0.69; 95% confidence interval (CI), 0.61–0.79; *p* < 0.001]. This association was insignificant among individuals with eGFR <30 mL min^−1^ 1.73 m^−2^ (*p* > 0.05). Our results suggested that preadmission statin therapy is associated with a lower incidence of AKI among critically ill patients; however, this effect might not be applicable for patients with eGFR <30 mL min^−1^ 1.73 m^−2^.

## 1. Introduction

Acute kidney injury (AKI) is defined as a rapid worsening of renal functions [1] and affects 2–18% of inpatients and 57% of critical care patients [2,3,4]. AKI in critically ill patients in the intensive care unit (ICU) is an important issue because it delays recovery and increases hospital mortality [5]. Thus, appropriately preventing AKI in the ICU is currently an important task in ICU patient management [6].

Statin, known as a 3-hydroxy-3-methylglutaryl-coenzyme A inhibitor, is one of the most commonly prescribed drugs worldwide [7] that lowers the risk of cardiovascular death by reducing the serum cholesterol level [8]. Furthermore, statin has anti-inflammatory, antithrombotic, and immunomodulating effects [9,10], also known as “pleiotropic effects” [11]. These pleiotropic effects are reported to lower the incidence of surgery-related [12,13], contrast-induced [14], and sepsis-related AKI [15]. However, some study findings show that statin failed to improve the outcomes of kidney disease, and the debate regarding the relationship between statin use and AKI is ongoing [16]. Thus, further studies are needed to substantiate the inhibitory effects of statin on AKI. Additionally, considering that statin therapy may be discontinued for many patients based on their states after ICU admission, it is important to clarify the association between preadmission statin use and AKI incidence after ICU admission.

This study aimed to investigate the association between preadmission statin use and AKI incidence after ICU admission in the general adult population. Additionally, we examined whether this association differs with respect to pre-ICU kidney function.

## 2. Materials and Method

This retrospective observational study was approved by the Institutional Review Board (IRB) of Seoul National University Bundang Hospital (IRB approval number: B-1806/474-105). Because of the retrospective nature of the study, the IRB waived the need to obtain informed consent from the patients. All data for the study were collected by a medical records technician who was blinded to the purpose of this study.

### 2.1. Patients

The medical records of adult patients aged ≥18 years who were admitted to the ICU between January 2012 and December 2017 were analyzed. When a patient was admitted to the ICU more than once during the study period, only data from the last ICU admission case, which might be the most severe, were included in the analysis. The exclusion criteria were as follows: (1) patients with an estimated glomerular filtration rate (eGFR; in mL min^−1^ 1.73 m^−2^) of <15 or those with end-stage renal disease (ESRD) who were undergoing renal replacement therapy (RRT) prior to admission because they usually received RRT after ICU admission regardless of AKI development; (2) patients lacking information on baseline creatinine or creatinine level within 72 h after ICU admission; and (3) patients diagnosed with AKI prior to ICU admission.

### 2.2. Preadmission Statin Use (Main Independent Variables)

Preadmission statin users were defined as patients who confirmed taking statins as maintenance treatment as prescribed by their physicians at least one month before ICU admission. The other cases were classified as non-statin users. Statin was classified as atorvastatin, rosuvastatin, simvastatin, pitavastatin, and other statins (pravastatin, fluvastatin, and lovastatin).

### 2.3. Measurements (Covariates)

Demographic information (sex, age, and body mass index) of the patients and comorbidities at ICU admission, including Acute Physiology and Chronic Health Evaluation II score and eGFR, total serum cholesterol at ICU admission (mg dL^−1^), and data regarding admission to the emergency department and other departments (internal medicine, neurologic center, cardiothoracic surgical department, and other surgical departments) were collected. Pre-ICU admission eGFR was computed using the Modification of Diet in Renal Disease formula [17]: eGFR (mL min^−1^ 1.73 m^−2^) = 186 × (creatinine level)^−1.154^ × (age)^−0.203^ × (0.742 if female). Using the cut-off points of total cholesterol as 160 and 200 mg/dL, which are known to be clinically meaningful, the subjects were divided into three groups (<160, 160–199, and ≥200) [18,19].

### 2.4. Diagnosis of AKI (Dependent Variable)

AKI was diagnosed based on the Kidney Disease: Improving Global Outcomes criteria and grading (Appendix A) [20]. Considering the varying lengths of urinary catheters used across patients, only serum creatinine (mg dL^−1^) was used for diagnosing AKI. Serum creatinine level measured at least within a month prior to ICU admission was defined as baseline creatinine, and AKI was diagnosed using serum creatinine levels measured within 72 h after ICU admission.

### 2.5. Outcomes

This study investigated how preadmission statin use is associated with the incidence of total AKI and stage ≥2 AKI after ICU admission. Additionally, we examined how this association differs according to preadmission eGFR.

### 2.6. Statistical Analysis

The baseline characteristics of the patients were presented as means with standard deviation or numbers with percentage. To compare preadmission statin users and non-statin users, continuous variables were tested using the two-sample t-test, while categorical variables were tested using the chi-square test. First, the individual association between each covariate and total AKI was examined with univariable logistic regression analysis. The covariates with *p* < 0.1 in the univariable logistic regression model were selected for adjustment in the final multivariable logistic regression analysis. Considering that baseline kidney function is a risk factor of AKI [21], we investigated the interaction between eGFR before ICU admission and preadmission statin use, and when there was an interaction, we performed a subgroup analysis by dividing the participants according to eGFR (≥90, 60–90, 30–60, and <30 mL min^−1^ 1.73 m^−2^). In the subgroup analysis, the Bonferroni correction was used to prevent type I errors that resulted from multiple comparisons [22]. The same method was used for analyzing stage ≥2 AKI as the dependent variable. All analyses were performed using IBM SPSS version 24.0 (IBM Corp., Armonk, NY, USA), and *p* < 0.05 was considered statistically significant.

## 3. Results

**Figure 1:**
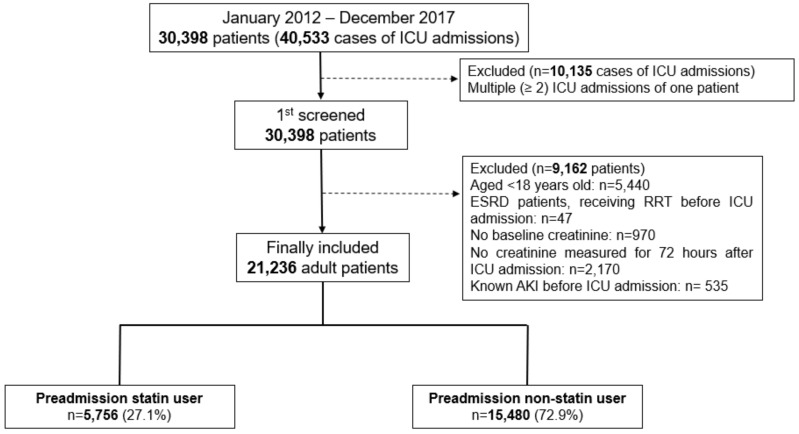
Flowchart of patient selection. ICU, Intensive Care Units; ESRD, End Stage Renal Disease; AKI, Acute Kidney Injury.

A total of 30,398 patients were admitted to the ICU 40,533 times between January 2012 and December 2017. These 30,398 patients were selected after excluding 10,135 cases involving the same patient being admitted to the ICU more than once. Next, we excluded 5440 patients aged <18 years, 47 ESRD patients who were undergoing RRT prior to ICU admission, 970 patients without baseline creatinine data, 2170 patients whose creatinine level was not measured within 72 h after ICU admission, and 535 patients who were diagnosed with AKI prior to ICU admission. As a result, 21,236 patients were included in the analysis, of whom 5756 (27.1%) were preadmission statin users and 15,480 (72.9%) were non-statin users (Figure 1). Their baseline characteristics are presented in Table 1. A total of 5469 (25.8%) patients developed AKI within 72 h after ICU admission, and 2216 (10.4%) of them had stage ≥2 AKI. Another 488 (2.3%) patients began postoperative RRT within 72 h after ICU admission.

### 3.1. Preadmission Statin Use and AKI Incidence

Table 2 shows the differences in characteristics between statin and non-statin users. The incidence of total AKI and stage ≥2 AKI among statin users was 1301/5756 (22.6%) and 439/5756 (7.6%), respectively, which was significantly lower than that in non-statin users [4168/15,480 (26.9%) and 1777/15,480 (11.5%), respectively] (*p* < 0.001). Table 3 shows the results of the multivariable logistic analysis after adjusting for the covariates selected in the univariate logistic regression analysis for total AKI incidence (Appendix B). AKI incidence within 72 h after ICU admission was 31% lower in preadmission statin users than in non-statin users [odds ratio (OR), 0.69; 95% confidence interval (CI), 0.61–0.79; *p* < 0.001]. Additionally, AKI incidence was 1.63-fold higher in patients with total cholesterol <160 mg dL^−1^ (OR: 1.63, 95% CI, 1.45–1.83; *p* < 0.001) than in those with total cholesterol of 160–200 mg dL^−1^ at ICU admission. There was no significant difference in patients with total cholesterol >200 mg dL^−1^ (*p* = 0.111).

An interaction occurred between eGFR before ICU admission and total AKI after ICU admission with respect to preadmission statin use (overall *p* = 0.001, in Table 3; model 1); thus, additional subgroup analysis was performed (Table 4). When the patients were divided according to eGFR at ICU admission, total AKI incidence within 72 h after ICU admission was 28% lower among statin users with eGFR ≥90 mL min ^−1^ 1.73 m^−2^ (OR, 0.72; 95% CI, 0.63–0.82; *p* < 0.001), 26% lower among statin users with 60 ≤ eGFR <90 mL min ^−1^ 1.73 m^−2^ (OR, 0.74; 95% CI, 0.61–0.91; *p* = 0.004), and 35% lower among statin users with 30 ≤ eGFR <60 mL min ^−1^ 1.73 m^−2^ (OR, 0.65; 95% CI, 0.51–0.83; *p* = 0.001) than among non-statin users. Meanwhile, there were no significant differences in total AKI incidence between groups with eGFR <30 mL min ^−1^ 1.73 m^−2^ (*p* = 0.095).

### 3.2. Preadmission Statin Use and Stage ≥2 AKI Incidence

Table 3 also shows the results of the multivariable logistic regression analysis for stage ≥2 AKI incidence, including the covariates selected in the univariable logistic regression analysis (Appendix C). Stage ≥2 AKI incidence within 72 h after ICU admission was 31% lower among preadmission statin users than among non-statin users (OR, 0.69; 95% CI, 0.57–0.84; *p* < 0.001; model 3). Additionally, stage ≥2 AKI incidence was 1.66-fold higher in patients with total cholesterol <160 mg dL^−1^ (OR: 1.66, 95% CI, 1.38–1.99; *p* < 0.001) than in those with total cholesterol of 160–200 mg dL^−1^ at ICU admission. There was no significant difference in patients with total cholesterol >200 mg dL^−1^ (*p* = 0.295). Moreover, no interaction occurred between eGFR at ICU admission and stage ≥2 AKI with resto preadmission statin use (overall *p* = 0.788).

## 4. Discussion

This study showed that preadmission statin use is associated with a lower incidence of AKI after ICU admission. This association was also evident with stage ≥2 AKI. However, the association was not significant among patients with severe kidney dysfunction (eGFR <30 mL min^−1^ 1.73 m^−2^) prior to ICU admission. Although the study results were derived from a retrospective observational study, it is striking because the statin group was comprised of significantly older and sicker patients and had a higher proportion of patients with renal dysfunction, more diabetes mellitus, ischemic heart disease, and cerebrovascular disease. Therefore, the study results suggested that clinicians who did not favor statin would consider prescribing statins to patients with respect to preventive effects for the development of AKI in critically ill patients.

The most interesting finding of this study was that an interaction occurred between eGFR at ICU admission and total AKI incidence with respect to preadmission statin use. In subsequent analyses, the potential benefit of preadmission statin use on AKI was not significant among patients with stage ≥4 chronic kidney disease (CKD). A meta-analysis, published in 2015, reported that statin therapy does not improve the overall kidney function of CKD patients with eGFR <60 mL min^−1^ 1.73 m^−2^ and that high-dose statin therapy leads to limited improvement in kidney function [23]. Another meta-analysis, published in 2017, concluded that statin therapy was not beneficial in reducing major cardiovascular events, cardiovascular death, and all-cause mortality of patients with CKD 4 or 5 (eGFR <30 mL min^−1^ 1.73 m^−2^) [24]. Although the primary endpoints were different from those used in our study, the previous meta-analysis suggested that statin therapy did not improve outcomes of patients with severe kidney dysfunction (CKD stage ≥4), which is consistent with our study finding. Patients with stage ≥4 CKD have worse baseline renovascular function than patients with normal kidney function and thus are more susceptible to ischemic oxidative damage, which is a major mechanism of AKI [25]. Furthermore, it was possible that treating patients with CKD stage ≥4 would be ineffective and the course of AKI could no longer be affected. However, it is difficult to completely explain the renal outcomes according to CKD stage solely based on this cohort study; hence, additional studies are needed.

This study suggested that preadmission statin therapy causes immunomodulatory effects, which were explained based on the pleiotropic effect of statin therapy [26]. We defined preadmission statin users as patients who confirmed taking statins as maintenance treatment as prescribed by their physicians at least one month before ICU admission. Most preadmission statin users received statin therapy for a long time, and there was some evidence that showed a clinical benefit of long-term statin therapy in patients with septic shock [27], pneumonia [28], or acute respiratory distress syndrome [29]. Although the immunomodulatory effect of statin therapy on critically ill patients remains controversial [30,31], it might affect the study results.

There is another important finding that should be carefully interpreted. The total incidence of AKI was higher in patients with <160 mg dL^−1^ of total serum cholesterol than in those with 160–200 mg dL^−1^ of total serum cholesterol, while patients with >200 mg dL^−1^ of total serum cholesterol had no association with the incidence of total AKI. In general, hyperlipidemia is an associated factor for renal damage [32]; however, hyperlipidemia was not associated with a lower incidence of AKI in this study. This can be explained based on the characteristics of ICU patients in this study. Lower cholesterol is a known factor that negatively affects the outcomes of critically ill patients [33,34], which is also coincident with our current study. Therefore, the effect of total serum cholesterol level on the incidence of AKI might be influenced by the characteristics of critically ill patients.

This study has a few limitations. First, a selection bias may have occurred due to the retrospective observational nature of the study. Second, the findings have limited generalizability because the study was conducted in a single center. For example, as previously mentioned, ethnical differences may have been involved in the effects of rosuvastatin. Lastly, because the duration of preadmission statin use differed among patients, we could not consider it in the analysis.

## 5. Conclusions

This study showed that preadmission statin use is associated with a lower incidence of total AKI and stage ≥ 2 AKI among critically ill patients after ICU admission. This association was most significantly evident among rosuvastatin users, but was absent among CKD patients with eGFR <30 mL min^−1^ 1.73 m^−2^.

## Figures and Tables

**Table 1:** Baseline characteristics of adults patients who were admitted to ICU in 2012–2017.

Variable		Total (21,236)	Mean	SD
Sex: male		12,434 (58.6%)		
Age, year			64.0	15.8
Body mass index, kg m^−2^			23.7	3.9
APACHE II			20.0	10.0
Comorbidities at ICU admission				
	eGFR ^a^ ≥ 90	12,993 (61.2%)		
	60 ≤ eGFR ^a^ < 90	4527 (21.3%)		
	30 ≤ eGFR ^a^ < 60	2364 (11.1%)		
	eGFR ^a^ < 30	1352 (6.4%)		
	Hypertension	9346 (44.0%)		
	Diabetes mellitus	1969 (9.3%)		
	Ischemic heart disease	538 (2.5%)		
	Cerebrovascular disease	945 (4.4%)		
	Chronic obstructive lung disease	921 (4.3%)		
	Liver disease (LC, hepatitis, fatty liver)	683 (3.2%)		
	Anemia (Hb < 10 g dL^−1^)	7569 (35.6%)		
	Cancer	4308 (20.3%)		
Characteristics of ICU admission				
	Admission through emergency department	12,042 (56.7%)		
	Admission department			
	Internal medicine	4671 (22.0%)		
	Neurologic center	4975 (23.4%)		
	Cardiothoracic surgical department	6875 (32.4%)		
	Other surgical department	4715 (22.2%)		
	Length of ICU stay, day		3.1	10.0
	Length of hospital stay, day		12.9	20.1
Preadmission statin use		5756 (27.1%)		
Total serum cholesterol at ICU adm, mg dL^−1^			138.2	47.9
	<160, mg dL^−1^	8584 (40.4%)		
	160–200 mg dL^−1^	10,751 (50.6%)		
	>200 mg dL^−1^	1901 (9.0%)		
Type of statin				
	Atorvastatin	3456 (16.3%)		
	Rosuvastatin	1391 (6.6%)		
	Simvastatin	396 (1.9%)		
	Pitavastatin	346 (1.6%)		
	Other statin ^b^	167 (0.8%)		
Total acute kidney injury		5469 (25.8%)		
Acute kidney injury stage ≥2		2216 (10.4%)		
RRT after ICU adm within 72 h		488 (2.3%)		

Presented as Number (percentage) or Mean value (standard deviation): ^a^: eGFR (mL min ^−1^ 1.73 m^−2^): 186 × (Creatinine)^−1.154^ × (Age)^−0.203^ × (0.742 if female); ^b^: Other statin: Pravastatin, Fluvastatin, and Lovastatin; ICU, intensive care unit; APACHE, acute physiology and chronic health evaluation; eGFR, estimated glomerular filtration rate; LC, liver cirrhosis; Hb, hemoglobin; RRT, renal replacement therapy.

**Table 2:** Comparison of characteristics between preadmission statin user and non-statin user.

Variables		Statin Group *n* = 5756	Non-Statin Group *n* = 15,480	*p*-Value
Sex: male		3398 (59.0%)	9036 (58.4%)	0.384
Age, year		68.6 (12.0)	62.2 (16.6)	<0.001
Body Mass Index, kg m^−2^		24.6 (3.8)	23.3 (3.8)	<0.001
Comorbidities at ICU admission				
	APACHE II	19.8 (9.8)	20.2 (10.1)	0.012
	eGFR ^a^			<0.001
	≥90	3073 (53.4%)	9920 (64.1%)	
	60–90	1488 (25.9%)	3039 (19.6%)	
	30–60	764 (13.3%)	1.600 (10.3%)	
	<30	431 (7.5%)	921 (5.9%)	
	Hypertension	3672 (63.8%)	5674 (36.7%)	<0.001
	Diabetes mellitus	830 (14.4%)	1139 (7.4%)	<0.001
	Ischemic heart disease	314 (5.5%)	224 (1.4%)	<0.001
	Cerebrovascular disease	505 (8.8%)	440 (2.8%)	<0.001
	Chronic obstructive lung disease	229 (4.0%)	692 (4.5%)	0118
	Liver disease (LC, hepatitis, fatty liver)	87 (1.5%)	596 (3.9%)	<0.001
	Anemia (Hb < 10 g dL^−1^)	1774 (30.8%)	5795 (37.4%)	<0.001
	Cancer	875 (15.22%)	3433 (22.2%)	<0.001
Admission through ED		2640 (45.9%)	9402 (60.7%)	<0.001
Admission department				<0.001
	Internal medicine	1317 (22.9%)	3354 (21.7%)	
	Neurologic center	1252 (21.8%)	3723 (24.1%)	
	Cardiothoracic surgical department	2166 (37.6%)	4709 (30.4%)	
	Other surgical department	1021 (17.7%)	3694 (23.9%)	
Total serum cholesterol at ICU adm, mg dL^−1^		125.2 (37.6)	143.1 (50.4)	<0.001
Length of hospital stay, day		11.3 (22.5)	13.5 (19.0)	<0.001
Length of ICU stay, day		2.5 (15.3)	3.3 (7.2)	<0.001
Total acute kidney injury		1301 (22.6%)	4168 (26.9%)	<0.001
Acute kidney injury stage ≥2		439 (7.6%)	1777 (11.5%)	<0.001
RRT after ICU adm within 72 h		140 (2.4%)	348 (2.2%)	0.426

Presented as number (percentage) or mean value (standard deviation). Two sample t-test for continuous variables and chi-square test for categorical variables were used: ^a^: eGFR (mL min ^−1^ 1.73 m^−2^): 186 × (Creatinine)^−1.154^ × (Age)^−0.203^ × (0.742 if female); ICU, intensive care unit; APACHE, acute physiology and chronic health evaluation; eGFR, estimated glomerular filtration rate; LC, liver cirrhosis; Hb, hemoglobin; ED, emergency department; RRT, renal replacement therapy.

**Table 3:** Multivariable logistic regression analysis for occurrence of acute kidney injury during 72 h after ICU admission.

Variable	Multivariable Model	
	Odds Ratio (95% CI)	*p*-Value
Dependent Variable: Total AKI		
Model 1: Preadmission statin use	0.69 (0.61, 0.79)	<0.001
Total serum cholesterol at ICU adm		
160–200 mg dL^−1^	1	<0.001
<160, mg dL^−1^	1.63 (1.45, 1.83)	<0.001
>200 mg dL^−1^	0.86 (0.72, 1.04)	0.111
Interaction: eGFR ^a^ ≥ 90 × Non-statin use	1	0.001
60 ≤ eGFR ^a^ < 90 × Statin use	1.19 (0.95, 1.49)	0.132
30 ≤ eGFR ^a^ < 60 × Statin use	0.97 (0.74, 1.26)	0.801
eGFR ^a^ < 30 × Statin use	1.89 (1.35, 2.65)	<0.001
Dependent Variable: Stage ≥2 AKI		
Model 3: Preadmission statin use	0.69 (0.57, 0.84)	<0.001
Total serum cholesterol at ICU adm		
160–200 mg dL^−1^	1	<0.001
<160 mg dL^−1^	1.66 (1.38, 1.99)	<0.001
>200 mg dL^−1^	0.85 (0.63, 1.15)	0.295
Interaction: eGFR ^a^ ≥ 90 × Non-statin use	1	
60 ≤ eGFR ^a^ < 90 × Statin use	0.95 (0.64, 1.40)	0.788
30 ≤ eGFR ^a^ < 90 × Statin use	1.04 (0.67, 1.61)	0.856
eGFR ^a^ < 30 × Statin use	1.27 (0.85, 1.91)	0.242

All covariates of *p* < 0.1 in univariable logistic regression analysis were included in multivariable logistic regression analysis. ^a^: eGFR (mL min ^−1^ 1.73 m^−2^): 186 × (Creatinine)^−1.154^ × (Age)^−0.203^ × (0.742 if female) ICU, intensive care unit; AKI, acute kidney injury; eGFR, estimated glomerular filtration rate.

**Table 4:** Multivariable logistic regression analysis for occurrence of acute kidney injury during 72 h after ICU admission according to eGFR at ICU admission.

Variable		Multivariable Model	
		Odds Ratio (95% CI)	*p*-Value *
eGFR ^a^ ≥ 90 (*n* = 12,993)			
	Preadmission statin use	0.72 (0.63, 0.82)	<0.001
60 ≤ eGFR ^a^ < 90 (*n* = 4527)			
	Preadmission statin use	0.74 (0.61, 0.91)	0.004
30 ≤ eGFR ^a^ < 60 (*n* = 2364)			
	Preadmission statin use	0.65 (0.51, 0.83)	0.001
eGFR ^a^ < 30 (*n* = 1340)			
	Preadmission statin use	1.33 (0.95, 1.86)	0.095

p * < 0.013 was considered as statistical significance after Bonferroni correction. ^a^: eGFR (mL min ^−1^ 1.73 m^−2^): 186 × (Creatinine)^−1.154^ × (Age)^−0.203^ × (0.742 if female). ICU, intensive care unit; eGFR, estimated glomerular filtration rate.

## Appendix A. Staging of Postoperative Acute Kidney Injury (KDIGO)

**Table:**

Stage	Serum Creatinine
1	1.5–1.9 times baseline or ≥0.3 mg dL^−1^ increase within 72 h after ICU admission
2	2.0–2.9 times baseline within 72 h after ICU admission
3	3.0 times baseline or increase in serum creatinine to ≥4.0 mg dL^−1^ or initiation of RRT within 72 h after ICU admission

KDIGO, Kidney Disease: Improving Global Outcomes; RRT, Renal Replacement Therapy.

## Appendix B. Univariable Logistic Regression Analysis of Covariates for Occurrence of Total Acute Kidney Injury during 72 h after ICU Admission

**Table:**

Variables			Odds Ratio (95% CI)	*p*-Value
Sex: male			1.06 (1.00–1.13)	0.066
Age, year			1.02 (1.02–1.02)	<0.001
Body mass index, kg m^−2^			0.96 (0.95–0.97)	<0.001
APACHE II			1.04 (1.04–1.04)	<0.001
Comorbidities at ICU admission				
	Hypertension		1.26 (1.18–1.34)	<0.001
	Diabetes mellitus		1.46 (1.32–1.61)	<0.001
	Ischemic heart disease		1.17 (0.97–1.42)	0.101
	Cerebrovascular disease		1.31 (1.14–1.51)	<0.001
	Chronic obstructive lung disease		1.13 (0.97–1.31)	0.109
	Liver disease (LC, hepatitis, fatty liver)		2.48 (2.13–2.89)	<0.001
	Anemia (Hb < 10 g dL^−1^)		3.82 (3.58–4.07)	<0.001
	Cancer		1.90 (1.77–2.05)	<0.001
	eGFR mL min^−1^ 1.73 m^−2^			
		≥90	1	<0.001
		60–90	0.99 (0.91–1.07)	0.731
		30–60	2.08 (1.89–2.28)	<0.001
		<30	5.18 (4.61–5.82)	<0.001
Admission through emergency department			1.52 (1.43–1.62)	<0.001
Total serum cholesterol at ICU adm				
160–200 mg dL^−1^			1	<0.001
<160, mg dL^−1^			2.21 (2.01, 2.43)	<0.001
>200 mg dL^−1^			0.81 (0.69, 0.94)	<0.001
Admission department				
	Internal medicine		1	<0.001
	Neurologic center		0.25 (0.23–0.28)	<0.001
	Cardiothoracic surgical department		0.78 (0.72–0.84)	<0.001
	Other surgical department		0.83 (0.76–0.90)	<0.001
Year at ICU admission				
	2012		1	<0.001
	2013		1.31 (1.16–1.48)	<0.001
	2014		1.26 (1.12–1.42)	<0.001
	2015		1.09 (0.97–1.23)	0.150
	2016		1.02 (0.91–1.15)	0.690
	2017		0.96 (0.86–1.08)	0.535

All covariates of *p* < 0.1 in univariable logistic regression analysis were included in multivariable logistic regression analysis; ICU, intensive care unit; AKI, acute kidney injury; APACHE, acute physiology and chronic health evaluation; LC, liver cirrhosis; Hb, hemoglobin.

## Appendix C. Univariable Logistic Regression Analysis of Covariates for Occurrence of Stage ≥ 2 AKI Acute Kidney Injury during 72 h after ICU Admission

**Table:**

Variables			Odds Ratio (95% CI)	*p*-Value
Sex: male			1.04 (0.95–1.13)	0.452
Age, year			1.01 (1.01–1.02)	<0.001
Body mass index, kg m^−2^			0.94 (0.92–0.95)	<0.001
APACHE II			1.04 (1.04–1.04)	<0.001
Comorbidities at ICU admission				
	Hypertension		1.08 (0.99–1.18)	0.106
	Diabetes mellitus		1.25 (1.09–1.44)	0.002
	Ischemic heart disease		0.94 (0.70–1.25)	0.654
	Cerebrovascular disease		1.03 (0.83–1.27)	0.795
	Chronic obstructive lung disease		0.97 (0.78–1.21)	0.816
	Liver disease (LC, hepatitis, fatty liver)		3.26 (2.73–3.88)	<0.001
	Anemia (Hb < 10 g dL^−1^)		4.86 (4.42–5.35)	<0.001
	Cancer		2.23 (2.03–2.46)	<0.001
	eGFR mL min^−1^ 1.73 m^−2^			
		≥90	1	<0.001
		60–90	0.73 (0.64–0.82)	<0.001
		30–60	1.10 (0.96–1.27)	0.171
		<30	2.84 (2.47–3.26)	<0.001
Admission through emergency department			2.00 (1.82–2.21)	<0.001
Total serum cholesterol at ICU adm				
160–200 mg dL^−1^			1	
<160, mg dL^−1^			2.34 (2.02, 2.70)	<0.001
>200 mg dL^−1^			0.81 (0.64, 1.04)	0.095
Admission department				
	Internal medicine		1	<0.001
	Neurologic center		0.20 (0.17–0.24)	<0.001
	Cardiothoracic surgical department		0.52 (0.46–0.58)	<0.001
	Other surgical department		0.67 (0.60–0.75)	<0.001
Year at ICU admission				
	2012		1	<0.001
	2013		1.52 (1.27–1.80)	<0.001
	2014		1.38 (1.16–1.63)	<0.001
	2015		1.19 (1.00–1.41)	0.053
	2016		1.05 (0.88–1.24)	0.605
	2017		1.00 (0.84–1.19)	0.999

All covariates of *p* < 0.1 in univariable logistic regression analysis were included in multivariable logistic regression analysis. ICU, intensive care unit; APACHE, acute physiology and chronic health evaluation; eGFR, estimated glomerular filtration rate; LC, liver cirrhosis; Hb, hemoglobin; RRT, renal replacement therapy.
